# Broccoli, Kale, and Radish Sprouts: Key Phytochemical Constituents and DPPH Free Radical Scavenging Activity

**DOI:** 10.3390/molecules28114266

**Published:** 2023-05-23

**Authors:** Camille Bowen-Forbes, Edward Armstrong, Audric Moses, Richard Fahlman, Helia Koosha, Jerome Y. Yager

**Affiliations:** 1 School of Public Health, University of Alberta, Edmonton, AB T6G 2R3, Canada; bowenfor@ualberta.ca (C.B.-F.); helia@ualberta.ca (H.K.); 2 Department of Pediatrics, Division of Neurology, Faculty of Medicine and Dentistry, University of Alberta, Edmonton, AB T6G 2R7, Canada; edwarda@ualberta.ca; 3 Lipidomics Core Facility, Faculty of Medicine and Dentistry, University of Alberta, Edmonton, AB T6G 2R7, Canada; audric.moses@ualberta.ca; 4 Department of Biochemistry, University of Alberta, Edmonton, AB T6G 2H7, Canada; rfahlman@ualberta.ca

**Keywords:** sulforaphane (SFA), sulforaphene (SFE), glucosinolates, erucic acid, antioxidant activity, total phenolics, cruciferous, broccoli, kale, radish

## Abstract

Our research group previously found that broccoli sprouts possess neuroprotective effects during pregnancy. The active compound has been identified as sulforaphane (SFA), obtained from glucosinolate and glucoraphanin, which are also present in other crucifers, including kale. Sulforaphene (SFE), obtained from glucoraphenin in radish, also has numerous biological benefits, some of which supersede those of sulforaphane. It is likely that other components, such as phenolics, contribute to the biological activity of cruciferous vegetables. Notwithstanding their beneficial phytochemicals, crucifers are known to contain erucic acid, an antinutritional fatty acid. The aim of this research was to phytochemically examine broccoli, kale, and radish sprouts to determine good sources of SFA and SFE to inform future studies of the neuroprotective activity of cruciferous sprouts on the fetal brain, as well as product development. Three broccoli: Johnny’s Sprouting Broccoli (JSB), Gypsy F1 (GYP), and Mumm’s Sprouting Broccoli (MUM), one kale: Johnny’s Toscano Kale (JTK), and three radish cultivars: Black Spanish Round (BSR), Miyashige (MIY), and Nero Tunda (NT), were analyzed. We first quantified the glucosinolate, isothiocyanate, phenolics, and DPPH free radical scavenging activity (AOC) of one-day-old dark- and light-grown sprouts by HPLC. Radish cultivars generally had the highest glucosinolate and isothiocyanate contents, and kale had higher glucoraphanin and significantly higher sulforaphane content than the broccoli cultivars. Lighting conditions did not significantly affect the phytochemistry of the one-day-old sprouts. Based on phytochemistry and economic factors, JSB, JTK, and BSR were chosen for further sprouting for three, five, and seven days and subsequently analyzed. The three-day-old JTK and radish cultivars were identified to be the best sources of SFA and SFE, respectively, both yielding the highest levels of the respective compound while retaining high levels of phenolics and AOC and markedly lower erucic acid levels compared to one-day-old sprouts.

## 1. Introduction

Cruciferous vegetables, including broccoli, kale, cauliflower, cabbage, and radish, have been shown to possess numerous health-promoting properties [1,2,3,4,5]. Our research group is particularly interested in the neuroprotective effects of broccoli sprouts (BrSps) during pregnancy. We found in rodent models of placental insufficiency and fetal inflammation that when BrSps are ingested during the last trimester of pregnancy, the effects on pathologic brain injury and the associated behavioral abnormalities are markedly reduced [6,7]. The active compound from BrSP has been identified as the isothiocyanate known as sulforaphane (SFA; 1-isothiocyanato-4-methylsulfinylbutane), obtained from hydrolytic action of the myrosinase enzyme on the glucosinolate (GSL), glucoraphanin (4-methylsulfinylbutyl glucosinolate; GRA), which is the dominant GSL in many broccoli varieties [8,9]. Glucosinolates are sulfur and nitrogen-containing glucosides that are found almost exclusively in cruciferous vegetables. When the plant tissues and cells are damaged, glucosinolates are hydrolyzed by the enzyme myrosinase, resulting in several pungent degradation products, including isothiocyanates (the most nutritionally and economically important products) and nitriles. Although the final product composition depends mainly on the chemical structure of the side chain, other factors such as pH and the presence of myrosinase-interacting proteins such as epithiospecifier protein (ESP) are also important [10]. Plants possessing ESP mainly produce nitriles and epithionitriles as hydrolysis products. These compounds have not been shown to be associated with beneficial health properties [11,12].

Isothiocyanates (ITCs) have been shown to possess powerful anticancer, antioxidant, anti-inflammatory, anti-diabetic, anti-microbial, anti-hypertensive, and neuroprotective properties [1,13,14,15,16]. They have been mostly studied in relation to their chemo-preventive properties. SFA has demonstrated potential in the treatment of conditions such as hypoxic-ischemic encephalopathy, neurodegenerative disease, hypertension, cancer, diabetes and autism spectrum disorder. Among its many modes of chemo-preventative action, upregulation of the phase-II enzymes, vis-à-vis the NrF2 pathway, and increase in the endogenous production of glutathiones—the most potent of the naturally occurring antioxidants, is the primary mechanism [17,18]. Kale is also known to be a good source of glucoraphanin and SFA. The structurally related isothiocyanate, sulforaphene (SFE; 1-isothiocyanato-4-methylsulfinylbut-1-ene) (predominantly found in radishes) has numerous benefits, some of which supersede those of SFA. Like SFA, SFE has demonstrated powerful chemo-preventive properties and is also a powerful phase-II enzyme inducer. In fact, SFA and SFE are often reported as the best natural products for cancer prevention [19].

**Figure 1:**
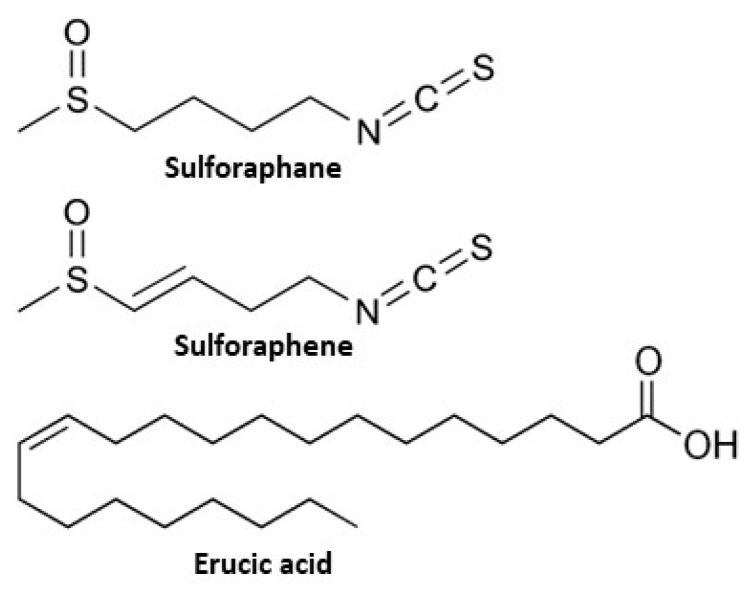
Structures of sulforaphane, sulforaphene, and erucic acid.

Although cruciferous seeds have higher levels of glucosinolates than sprouts and therefore have greater potential to induce phase II detoxication enzymes, they also have high levels of erucic acid (22:1ω9), an antinutritional fatty acid whose concentration decreases with sprouting [20]. Negative health impacts associated with erucic acid include myocardial lipidosis, heart lesions, and impaired oxidative phosphorylation [20,21]. If cruciferous materials are to be used in health-related research, it is critical to consider the levels of not only the beneficial constituents, but also those of the potentially harmful constituents. The structures of SFA, SFE, and erucic acid are presented in Figure 1.

Whereas SFA and SFE are the primary active compounds found in broccoli and kale (SFA), and radishes (SFE), it is probable that other constituents, such as phenolics, contribute to their activity and also play a role in their beneficial effects. Research has shown that concentrated mixtures of glucoraphanin and myrosinase are not as effective in up-regulating antioxidant genes as natural whole broccoli sprouts, which is likely due to synergistic effects between sulforaphane and other phytochemicals. Phenolics are powerful antioxidants that are ubiquitous to plants and are present in high levels in crucifers. They act mainly via free radical scavenging and metal chelation. In addition to these effects, molecular studies have revealed that phenolics can exert modulatory actions in the cellular environment by interacting with a wide spectrum of molecular targets central to the cell signaling machinery, including activation of phase II antioxidant detoxifying enzymes [22,23,24].

The aim of this study was to evaluate key phytochemical components of seven cultivars of broccoli, kale, and radish sprouts in order to guide further studies of the neuroprotective activity of cruciferous sprouts on fetal and newborn brains. Specific cultivars were chosen for study either because they were identified to be good sources of key glucosinolates or isothiocyanates or based on availability. Accordingly, the cultivars studied were Johnny’s Sprouting Broccoli (JSB), Gypsy F1 broccoli (GYP), Mumm’s Sprouting Broccoli (MUM), Johnny’s Toscano Kale (JTK), and the radish cultivars: Black Spanish Round (BSR), Miyashige (MIY), and Nero Tunda (NT). Cultivars were allowed to germinate for one, three, five, and seven days and were tested in order to determine the most suitable sprouting duration that yields high levels of the desired phytochemicals.

We first quantified the GSL and phenolics content of the sprouts in order to determine their potential for yielding high levels of ITCs and phenolics in the hydrolyzed extracts. The erucic acid contents of the sprouts were also determined, as high levels of this compound would not be desirable for this purpose. Subsequently, we determined the ITC, DPPH antioxidant activity, and phenolic levels of the hydrolyzed extracts. One-day-old sprouts were used for preliminary testing, as opposed to seeds, as the glucosinolates (and phenolics) contents are known to decrease markedly (and to varying extents from one cultivar to the next) upon germination [25,26,27,28]. One-day-old sprouts, therefore, give a better idea of how the levels of these compounds will change upon further sprouting. Based on the results, selected cultivars of each vegetable type were chosen for more prolonged sprouting (up to seven days) to determine how the levels of the various constituents of interest changed over time. Principal component analysis (PCA) and Pearson’s correlation coefficient were used to analyze the data. PCA is a statistical tool that enables the visualization of the interrelationships between the parameters analyzed and the cultivars studied [29]. PCA was used to determine the relationship between the various phytochemicals analyzed. In addition to numerical correlation data, the relationships were classified according to a color scale, with blue indicating a positive correlation and red, a negative correlation. Color intensity indicated the strength of the correlation. This research will provide useful information for guiding further studies on the effects of cruciferous sprouts on health, as well as product development.

## 2. Results

### 2.1. Analysis of One-Day-Old Sprouts

#### 2.1.1. Glucosinolates Contents

**Figure 2:**
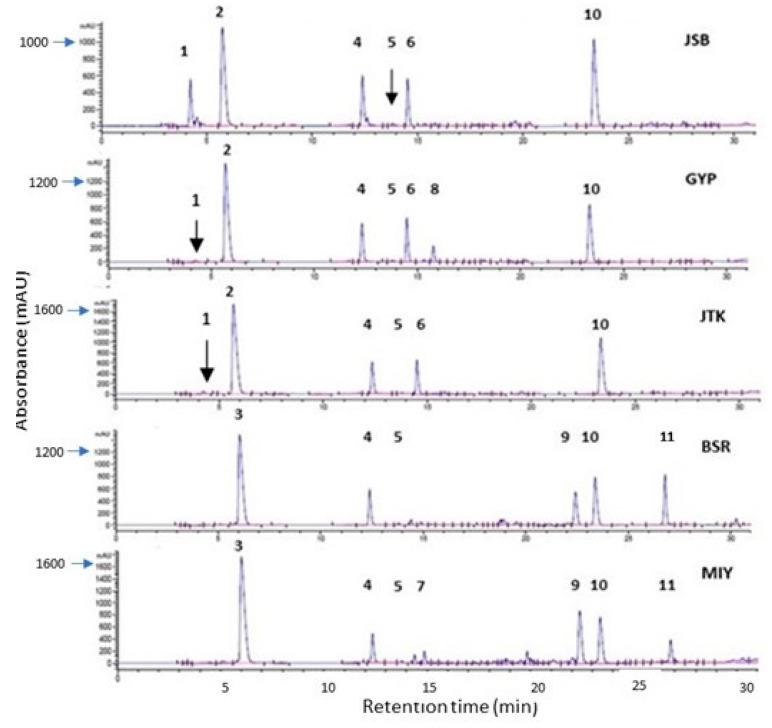
Chromatograms of selected one-day-old broccoli, kale, and broccoli cultivars (227 nm) showing glucosinolates and phenolic compounds. Broccoli: Johnny’s Sprouting Broccoli (JSB), Gypsy F1 (GYP); Kale: Johnny’s Toscano Kale (JTK); Radish: Black Spanish Round (BSR), and Miyashige (MIY). Compounds: 1: Glucoiberin; 2: glucoraphanin; 3: glucoraphenin; 4: 4-hydroxyglucobrassicin; 5: glucoiberverin; 6: glucoerucin; 7: glucoraphasatin; 8: glucobrassicin; 9–11: Unidentified phenolics.

The radish cultivars (BSR, MIY, and NT) possessed the same general GSL profile (Figure 2), containing glucoraphenin (GRE), glucoraphasatin (GRPH), 4-hydroxy glucobrassicin (4OHGBR), and glucoiberverin (GIV) (in order of concentration) as the major GSLs. This is consistent with the literature, with GRE commonly being identified as the major GSL in radish seeds and sprouts [25,30,31]. With the exception of the more noted glucoiberin (GIB) for JSB and MUM, the kale and broccoli cultivars had the same general GSL profile, containing GRA, GER, 4OHGBR, GIB, GIV, and GBR, in order of concentration. This similarity between the kale and broccoli cultivars is not surprising, as both crucifers are classified botanically as different varieties of the same plant species [32]. The finding of GRA as the major glucosinolate in broccoli and kale is consistent with the literature [10,26]. There was no significant difference among the glucosinolate contents of one-day-old sprouts grown under dark and light conditions. Similar to our findings, Vale et al. found non-significant differences in kale and broccoli sprouts (seven and nine-day-old) grown under light and dark conditions [27]. On the other hand, another study found significantly lower levels of glucosinolates in broccoli sprouts (three, five, and seven days old) grown in the dark compared to those grown in the light [26]. Ciska and coworkers found that the effect of lighting conditions on GSL levels in one-day-old radishes changed with variety, with one-day-old red radishes yielding higher levels, and the reverse is true for white radishes. However, the light-grown sprouts harvested on days three–six had greater GRE contents. As was the case in the present study, Ciska found that sprouts that germinated in the dark had higher total indole contents characteristically. Higher total GSL levels were also reported for dark-grown radishes over a one–seven-day harvesting period [28]. A limitation of this study is the short sprouting duration used to compare the effects of lighting conditions on the levels of glucosinolates and other phytochemicals. As there were no significant differences observed for different lighting conditions, the results obtained for dark-grown sprouts are discussed hereafter.

**Figure 3:**
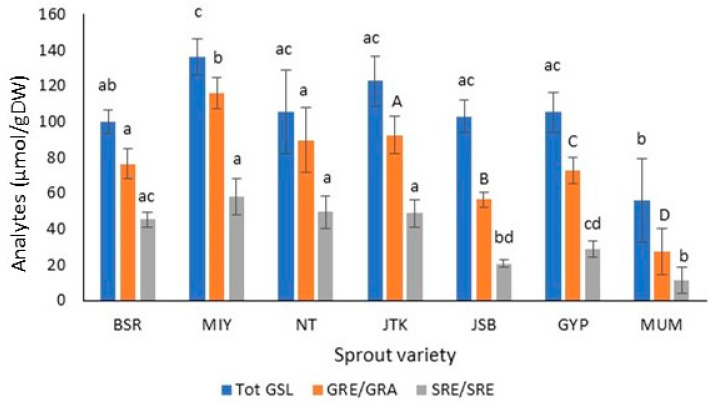
Total glucosinolates (Tot GSL), glucoraphenin (GRE), glucoraphanin (GRA), sulforaphene (SFE), and sulforaphane (SFA) levels in dark-grown radish, kale, and broccoli cultivars. (For the ITC of JSB, light-grown sprouts are used.) Broccoli: Johnny’s Sprouting Broccoli (JSB), Gypsy F1 (GYP) Mumm’s Sprouting Broccoli (MUM); Kale: Johnny’s Toscano Kale (JTK); Radish: Black Spanish Round (BSR), and Miyashige (MIY), Nero Tunda (NT). GRE and SRE are in radishes only; GRA and SFA are in kale and broccoli (n = 3; ±SD). For a given analyte type, values followed by the same letter and font type are not significantly different (*p* ≤ 0.05). Upper-case letters are used for GRA only.

MIY had significantly greater levels of GRE than the other two radish cultivars and had the highest total GSL content (136.21 µmol/g DW), which was significantly higher than those of BSR radish and MUM broccoli cultivars (89.91 and 56.11 µmol/g DW, respectively). (Table 1, Figure 2 and Figure 3). JTK had the highest level of GRA (92.57 µmol/g DW) among the kale and broccoli cultivars, while MUM had the lowest level (27.59 µmol/g). The same trend was seen for the total GSL levels. Vale et al. also found that Galega kale sprouts (seven-and-nine-day-old) had greater GSL levels than broccoli sprouts at the same harvesting time points [27]. With a GSL content of 107.92 µmol/g DW, JSB had the highest GSL content of the broccoli cultivars analyzed. GIV and the indole GSL, 4OHGBR, were present in all seven cruciferous cultivars studied, 4OHGBR being present within a narrow range of 4.42 µmol/g DW (MUM) to 5.91 µmol/g DW (JSB). It is known that 4OHGBR is among the indole glycoside in broccoli, kale, radish seeds, and sprouts [9,20,27]. Glucobrassicin, the other indole GSL, was detected in the kale and broccoli sprouts (0.61–1.89 µmol/g DW) but not in the radish sprouts. There was no significant difference in the total indole GSL among the sprouts analyzed, with levels ranging from 4.88 µmol/g DW (MIY) to 6.67 µmol/g DW (GYP). Kale and broccoli sprouts generally had higher total indole GSL levels compared to radish sprouts. Most GBR breakdown products are known to induce the synthesis of phase one detoxifying enzymes, which may prevent carcinogenesis in some cases; however, in other cases, they can promote carcinogenesis [33]. Glucobrassicin conversion in the stomach can produce oligomeric compounds that may potentially be very dangerous to human health, and it is believed that analogous oligomeric compounds may be produced by 4OHGBR [20] and possibly other indole GSLs. Because of this, high levels of indole GSLs are not generally desirable.

#### 2.1.2. Isothiocyanates in Sprout Extracts

**Figure 4:**
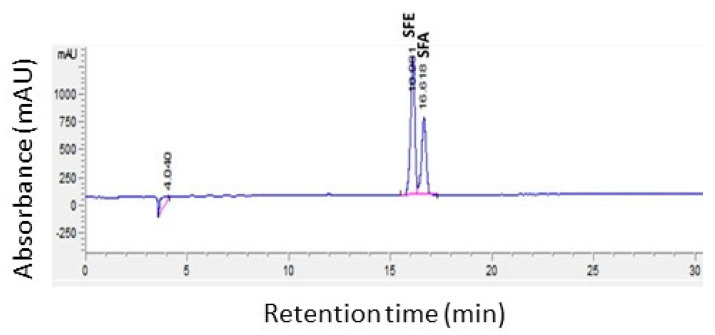
HPLC Chromatogram of a mixture of sulforaphene (SFE: t_R_16.08 s) and sulforaphane (SFA: t_R_16.62 s) at 205 nm.

The radish cultivars generally had the highest levels of ITC (45.57–58.23 µmol/g DW), followed by kale (48.76 µmol/gDW) and broccoli cultivars (11.25–28.96 µmol/g DW) (Figure 3). There was no significant difference among the ITC levels of the radish and kale varieties, which were significantly higher than those of the broccoli varieties. MUM sprouts had significantly lower ITC levels than the other two broccoli cultivars. A very strong correlation existed between the total GSL and the SFE or SFA contents (r = 0.82). There was also a very strong positive correlation (r = 0.95) between the GRE (radish) and GRA (kale and broccoli cultivars) and their respective SFE and SFA levels (Table 2), with average GSL:ITC conversions of 55 and 43% in radishes and broccoli, respectively, and 41% in the kale cultivar analyzed. This higher percent conversion observed in radish sprouts is not surprising, considering the presence of ESP enzyme in broccoli and kale and the absence in radishes. Interestingly, it has been found that heating decreases ESP activity and results in increased SRA formation in broccoli florets and sprouts. Matusheski et al. (2004) showed that heating fresh florets to 60 °C for 5 min markedly reduced ESP activity and increased the extraction yield of SRA. The same effect was seen when fresh sprouts were heated to 70 °C for 10 min [34,35,36]. The difference in heat lability of these two enzymes may be exploited in food product development. Figure 4 shows a chromatogram of a resolved mixture of SFE and SFA.

#### 2.1.3. Phenolics Contents and Antioxidant Capacity

**Figure 5:**
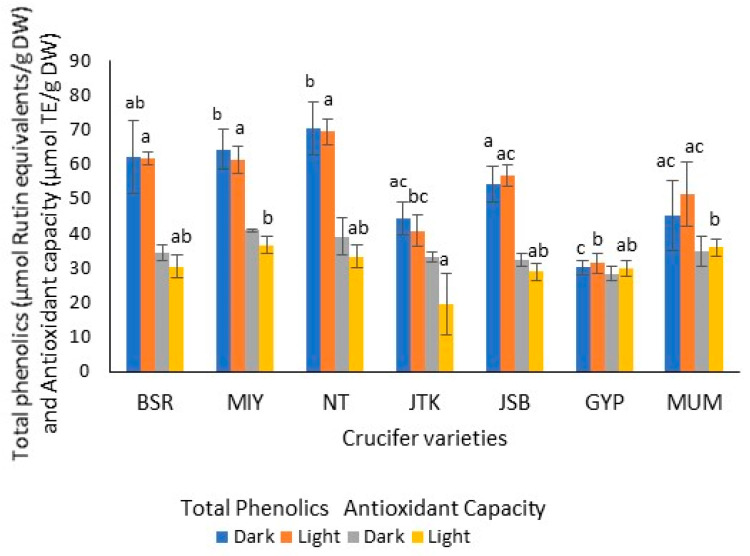
Total phenolics (µmol Rutin equivalents/g DW) and antioxidant capacity (DPPH radical scavenging activity: µmol Trolox equivalents/g DW) of one-day-old sprout extracts grown under dark and light conditions. Radish: Black Spanish Round (BSR), Miyashige (MIY), and Nero Tunda (NT); kale: Johnny’s Toscano Kale (JTK); broccoli: Johnny’s Sprouting Broccoli (JSB), Gypsy F1 (GYP), and Mumm’s sprouting broccoli (MUM). (n = 3; ±SD). For a given analyte and lighting condition, values followed by the same letter are not significantly different (*p* ≤ 0.05). For all the sprout varieties, there was no significant difference observed in the analyte levels for different lighting conditions.

As was observed for the GSL analysis, the three radish cultivars had the same general phenolics profile, and the same was true for the kale (JTK), JSB, and GYP broccoli cultivars. All seven cultivars tested had the same major phenolic compound (t_R_ = 23.29 min), which, though unidentified, had the same mass spectral characteristics; the radish cultivars had higher levels of phenolics than kale and broccoli. Dark-grown radish and kale sprouts had slightly higher phenolics contents than their light-grown counterparts, while the converse was true for broccoli cultivars (Figure 5). Nonetheless, there was no significant difference among these levels at the 95% confidence level. For dark-grown sprouts, NT had the highest levels (70.41 µmol/g DW). JSB had the highest phenolics levels of the kale and broccoli cultivars (54.40 µmol/g DW), while GYP had the lowest level (30.15 µmol/g DW).

Lighting conditions did not significantly affect the DPPH inhibitory activities (antioxidant capacities) of the one-day-old crucifer sprouts. Among the dark-grown sprouts, there was no significant difference in AOC across the seven cultivars (Figure 5). While light-grown radishes had higher AOC than the other similarly grown cultivars, this was only significant relative to the kale cultivar. In contrast to our findings, Vale et al. found that the antioxidant activity of Brassicaceae sprouts (broccoli, radish, etc.) was significantly greater when growth occurred under light compared to dark conditions [37].

AOC had a moderately positive correlation to phenolic contents (r = 0.61). It is well established that phenolic compounds are good electron donors, which is their primary mechanism of action. In addition, some phenolics stimulate the synthesis of endogenous antioxidant molecules within the cellular environment [38,39]. One study found that the phenolic content of Brassica sprouts, including broccoli, cabbage and kale cultivars, contributed significantly to the antioxidant capacity [37]. It was revealed in a study on nine Brassica vegetables that myricetin, quercetin-3-galactoside, and quercetin-3-glucoside were the three most potent antioxidant compounds in the species analyzed [40]. Li and coworkers (2018) found that the antioxidant activity of 12 cruciferous vegetables ranged from 1.11 to 9.54 µmoles Trolox equivalent/g FW. This value is notedly less than what we found in this study, which is no doubt attributable to their use of metrics relative to fresh weight, compared to our use of dry weight for our calculations, as well as the difference in plant maturity (i.e., mature versus sprouts) [41].

Interestingly, there was a strong negative correlation between both phenolics contents and AOC with indole glycoside levels (r = −0.71 and −0.72, respectively; Table 2).

#### 2.1.4. Fatty Acid Content

Erucic acid was the most dominant fatty acid in all the sprout samples tested, apart from BSR and NT, which had slightly higher levels of the monounsaturated fatty acid, oleic acid (18:1c9). In addition to oleic acid, linoleic (18:2), α-linolenic (18:3ω3), eicosenoic (20:1ω15), and palmitic (16:0) acids were also present in appreciable amounts in all the cultivars analyzed (Table 3). Seeds of 20 accessions of six Brassica species contained oleic, linoleic, linolenic, erucic, palmitic, and stearic acids as the major fatty acids [42], similar to our findings. Radish sprouts were characterized by the highest fatty acid content (approximately 61.36–65.75 mg fatty acid/g DW), compared to 49.55 mg/g DW in kale and 45.76–48.46 mg/g DW in broccoli cultivars (Table 4). A study of 59 cruciferous seeds found that erucic acid ranged from 27.0–56.7% in hexane-extractable lipids [20]. This agrees closely with our finding of approximately 25–50% erucic acid in the lipid extract of the one-day-old cruciferous sprouts (Table 2).

As erucic acid (22:1) is a fatty acid of health concern, the discussion here will be mainly focused on this analyte. In contrast to what was observed for the total fatty acids, radish sprouts generally had the lowest erucic acid levels (16.28–18.89 mg/g DW), compared to 23.48 mg/g DW for the kale and 17.83–23.06 mg/g DW for the broccoli sprouts (Table 4). All seven sprout cultivars produced greater amounts of fatty acids when grown in the light compared to the dark. Similarly, higher levels of erucic acid were observed in all the light-grown cultivars (Table 4). Lighting conditions, however, did not affect the proportion of FAs produced by the sprouts (Table 3). For dark-grown sprouts, radish cultivars had the lowest percentage of erucic acid (24.76 to 30.80%) in the fatty acid extract, followed by broccoli (39.16 to 49.44%) and kale (47.41). Although all seven cultivars had high levels of monounsaturated acids, which are usually considered to be “healthy fats”, a significant proportion of this is attributable to erucic acid, which—although common among cruciferous vegetables—is not characteristic of most other vegetable oils. BSR and NT had lower erucic acid contents than MIY radish, and GYP had the lowest erucic acid level of the broccoli sprouts. Kale had the highest erucic acid content of all seven cultivars. With the exception of the moderate positive correlation between erucic acid and indole GSL (r = 0.51), erucic acid correlated negatively with all parameters analyzed, this being strong for phenolics levels. Interestingly, indole GSL correlated negatively with all analytes except erucic acid (Table 2). A study on *Brassica rapa* (turnip) seeds showed that there was a significantly positive correlation between the contents of erucic acid and glucosinolates [43]. We observed a weak negative correlation (−0.37) between these two analytes in our study.

#### 2.1.5. Identification of Cultivars for Study on Effect of Sprouting Times on Different Analytes

Based on the phytochemistry of the one-day-old cultivars, the best potential source of SFA among the broccoli and kale cultivars tested was determined to be JTK (dark-grown). Furthermore, kale sprouts had the highest GRA content and the second-highest phenolics content. However, JSB (light-grown), which is much less expensive than JTK, becomes a more favourable option when economic costs associated with scaled up research and development are considered: It costs almost three times less to buy JSB seeds compared to JTK seeds [44,45], and JSB sprouts possess 64% of the GRA levels in JTK sprout. Additionally, JTK has higher erucic acid content than JSB. (Of note, NT possessed only slightly less GSL and markedly greater phenolics contents compared to MIY.) Although MIY had the highest GRE level of the cultivars tested, it had the lowest GRE:SFE conversion factor (50%). On the other hand, BSR had the lowest GRE level but had the highest conversion factor (60%). Although MIY was the highest-yielding source of SFE of the radish cultivars, there was no significant difference among their SFE levels. Furthermore, BSR seeds are much less expensive than MIY seeds (approximately by a factor of five) [46,47], while having over 70% of the GSL contents of MIY and approximately 80% of the phenolics contents. Considering the foregoing, JSB and BSR were chosen for analysis of the effect of sprouting times on the various parameters of interest. JTK, the lone kale variety, was also included.

### 2.2. Analysis of Three-to-Seven-Day-Old Sprouts

#### 2.2.1. Glucosinolates in Three-, Five-, and Seven-Day-Old Sprouts

The three, five, and seven-day-old radish (BSR), kale (JTK), and broccoli (JSB) cultivars analyzed contained the same GSL as those in the respective one-day-old sprouts, with the exception of progoitrin (PRO), which was not detected in the latter sprouts. There were, however, changes in the GSL levels with time (Table 5). BSR harvested on day five had higher GSL levels than three- and seven-day-old sprouts. GRE and GRPH were the two major GSLs in BSR. The levels of GRE decreased from 32.78 to 8.34 µmol/g DW in going from day three to day seven of harvesting, an almost four-fold decrease. On the other hand, the GRPH level was highest for five-day-old sprouts (50.30 µmol/g DW) and lowest for seven-day-old ones (13.06 µmol/g DW). Three- and seven-day-old JTK sprouts had significantly higher total GSL levels than five-day-old sprouts. Not surprisingly, the GRA levels in three- and seven-day-old JTK sprouts (78.83 and 77.09 µmol/g DW, respectively) were also significantly higher than that of sprouts harvested after five days. A similar trend was seen for glucoerucin, albeit the levels were markedly lower than GRA (3.88–15.83 µmol/g DW). For JSB sprouts, the levels of all the GSLs decreased with harvesting time except for GIV and PRO, which had the highest levels in five-day-old sprouts. Previous research found that Galega kale sprouts had higher glucosinolate content (mainly sinigrin and glucoiberin) compared to broccoli and cabbage varieties. The GSL levels decreased over time, being higher for seven- and nine-day-old sprouts than for 12-day-old sprouts [27]. In summary, over the three–seven-day harvesting period studied, three-and seven-day-old JTK sprouts had the highest total GSL levels (109.85 and 105.47 µmol/g DW, respectively), with the associated highest level of GRA (78.83 and 77.09 µmol/g DW, respectively). The levels of total GSLs as well as the GRA and GRE levels (in JSB and BSR, respectively), were similar for JSB and BSR, with the exception of BSR harvested after five days, which had higher GSL levels, due to the high level of GRPH at that stage. Three-day-old BSR and three- and seven-day-old JTK were therefore found to be the best respective sources of GRE and GRA for the harvesting period studied.

#### 2.2.2. Determination of Isothiocyanate Levels

**Figure 6:**
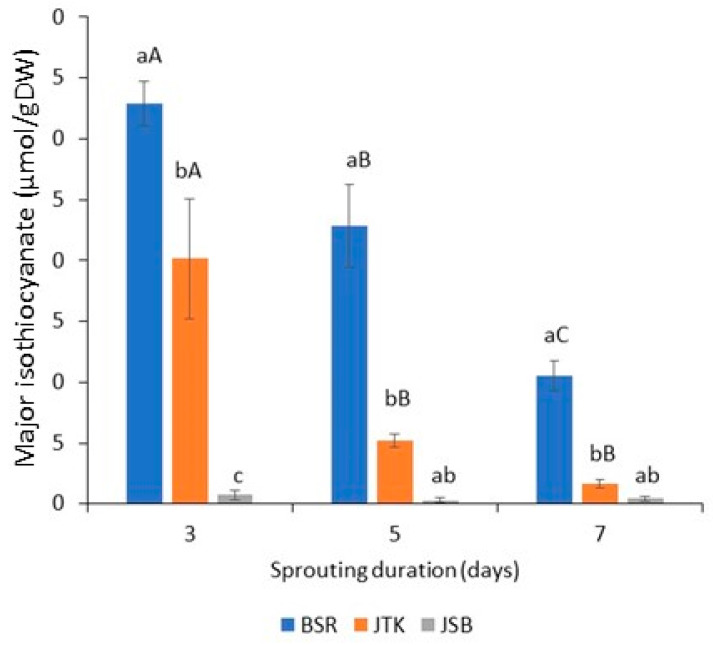
Content of major isothiocyanate in three-, five-, and seven-day radish, kale, and broccoli cultivars: sulforaphene is found in Black Spanish Round (BSR) radishes, and sulforaphane is found in Johnny’s Toscano Kale (JTK) and Johnny’s Sprouting Broccoli (JSB); (n = 3; ±SD). Different lowercase and upper-case letters above bars, respectively, indicate significant differences for samples of the same sampling day and samples of the same variety at *p* < 0.05, according to Tukey’s Multiple Comparison Test.

Although the highest GSL levels were observed for kale sprouts, this trend was not reflected in the ITC levels. Rather, the radish sprouts had significantly higher levels of ITC than both kale and broccoli sprouts over the three–seven-day sprouting duration, with the levels decreasing with time for all crucifer types (Figure 6). Kale sprouts had higher ITC levels than broccoli sprouts for all three sprouting times, and this was significant at the three-day time point. The relatively high levels of SFE found in the radish, compared to the SFA found in broccoli and kale cultivars, is likely to be primarily attributed to the fact that, in addition to myrosinase, kale and broccoli contain epithiospecifier enzyme (EPI), which produces epithionitriles from GSLs, effectively lowering the GSL:ITC conversion. Radishes, on the other hand, are devoid of EPI and so have a higher GSL:ITC conversion rate, as there is no competition for myrosinase from EPI. Vale and coworkers previously found that Galega kale had higher GSL contents and myrosinase activity compared to broccoli and cabbage sprout cultivars (in particular, seven- and nine-day-old sprouts) [27].

#### 2.2.3. Determination of Phenolics Levels and Antioxidant Capacity (AOC)

**Figure 7:**
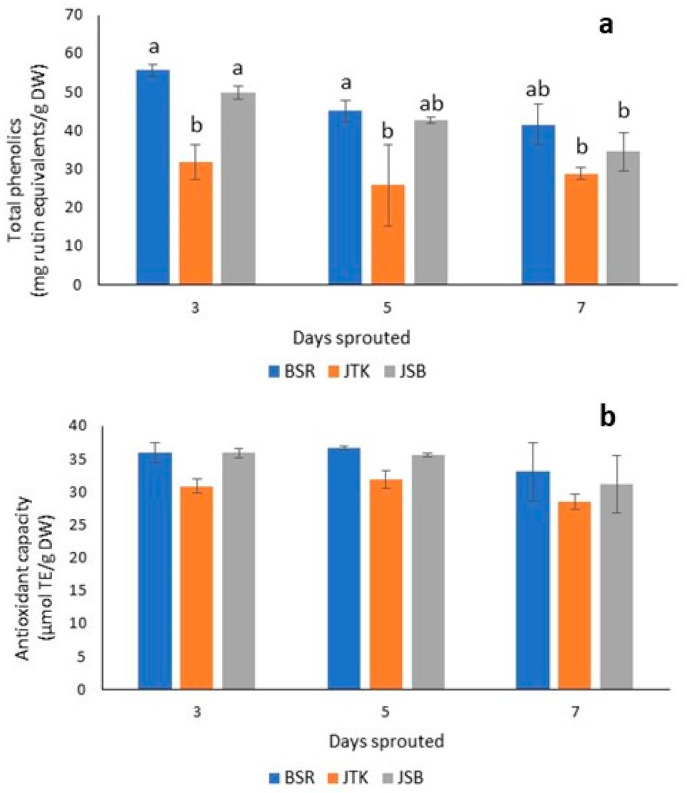
Total phenolics (uM rutin equivalents/g DW) (**a**) and DPPH radical scavenging activity (µmol TE/g DW) (**b**) of three-, five-, and seven-day-old sprout extracts. Black Spanish Round radish (BSR); Johnny’s Toscano Kale (JTK); Johnny’s Sprouting Broccoli (JSB). Different letters above bars indicate significant differences at *p* < 0.05 according to Tukey’s Multiple Comparison Test.

BSR sprouts had the highest phenolics contents over the three–seven-day sprouting duration, with the levels in BSR and JTK being significantly greater than that in JSB (Figure 7). The AOCs of the sprout extracts were not significantly different over the sprouting duration or across varieties, and the values also agreed closely with those of one-day-old sprouts (Figure 5). Sprouting time, therefore, did not have a significant effect on AOC in general. The same trend observed for AOC was seen for total phenolics levels in the cruciferous sprouts analyzed, with no significant difference observed in any instance. Although the radish and kale sprouts had a higher AOC than the broccoli sprouts, neither variety nor sprouting time significantly impacted AOC.

#### 2.2.4. Determination of Fatty Acid Contents

Although the proportion of erucic acid in the three cultivars analyzed (BSR, JTK, and JSB) did not change notably with harvesting time, there was a marked decrease in the total fats produced by the sprouts over time (Table 6 and Table 7), with a resultant decrease in erucic acid contained in the respective sprouts. Relative to the total fatty acid content of one-day-old sprouts, the total fatty acid contents of the three cultivars decreased by approximately 50% by day seven of harvesting (Table 4 and Table 7). As was the case for the one-day-old sprouts, three–seven-day-old kale sprouts had the highest erucic acid levels (12.23–18.35 mg/g DW for seven- and three-day-old sprouts, respectively). The radish sprouts, however, had higher total fatty acid contents.

#### 2.2.5. Principal Component Analysis (PCA) and Pearson Correlation Coefficient

**Figure 8:**
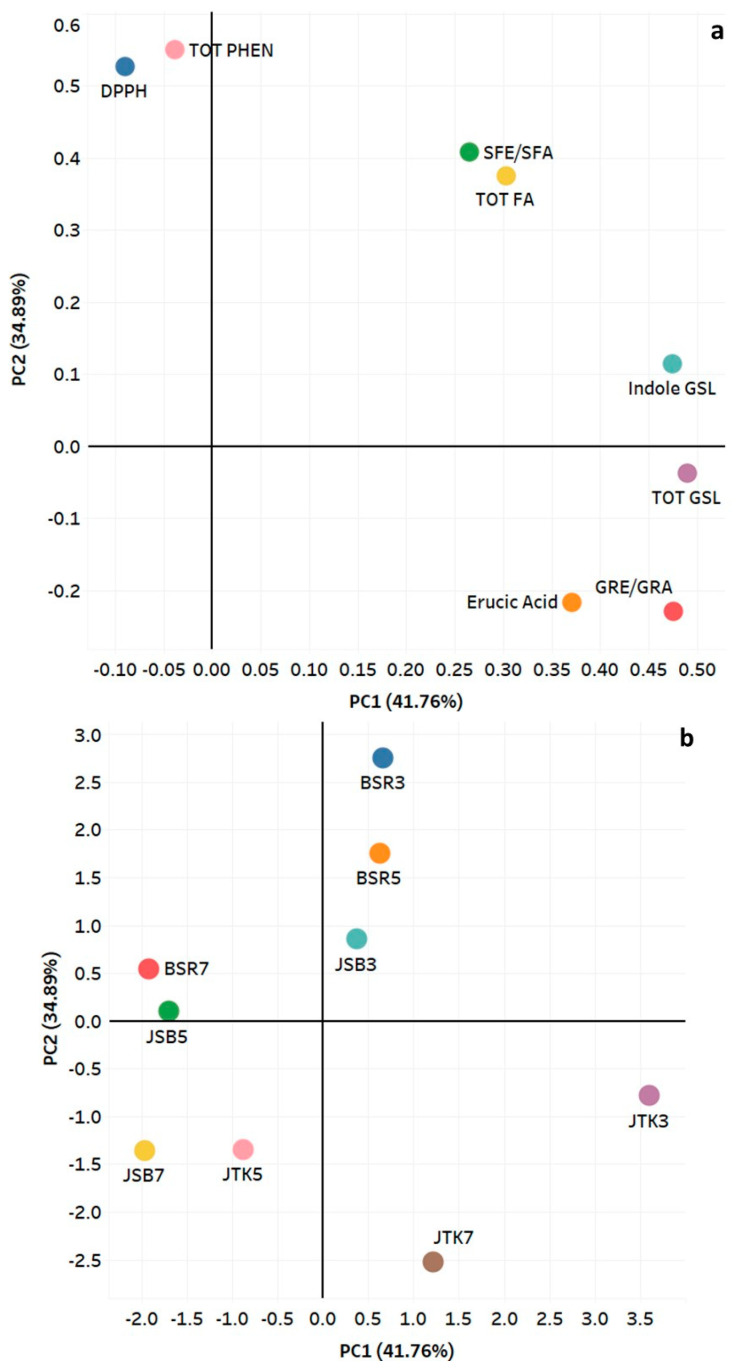
Principal component analysis showing: (**a**) correlation of phytochemical parameters with PC1 and PC2, and (**b**) relationship of three cruciferous cultivars at three harvest dates. PCA was performed on the correlation matrix of average values of phytochemical parameters and antioxidant capacity (DPPH).

Principal component analysis (PCA) is one of the most notable dimensionality reduction techniques used in research [48,49]. In PCA, the measured variables are transformed into new uncorrelated variables called principal components. The first component describes most of the variation in the data. The second principal component, which is orthogonal, accounts for much of the remaining variation [29]. PCA was performed on the analytes of focus for this study, namely: glucoraphanin and sulforaphane (for broccoli and kale), glucoraphenin and sulforaphene (for radish), total indole glycosides, erucic acid, total fatty acids, total phenolics, and DPPH free radical scavenging activity (Figure 8). After performing PCA, based on the resulting scree plot and the eigenvalues of the correlation matrix, only the first two principal components (PCs) were selected as principal components of interest, both of which gave eigenvalues greater than zero (3.34 and 2.79, respectively). PC1 explains 41.76% of the variation found in the data, and PC2 explains 34.89% of the data’s variation. Cumulatively the two explain 76.65% of the variation found in this data.

Figure 8 shows the interrelation among the phytochemical parameters analyzed (a) the positioning of the cultivars studied in comparison to each other (b). By examining the generated eigenvectors matrix for PC1 and PC2, it was determined that the four variables GRE/GRA, total GSL, indole GSL, and erucic acid load heavier on PC1 while the other four variables SFE/SFA, total fatty acids, and total phenolics, and DPPH radical scavenging activity load heavier on PC2. The predominant phytochemicals in PC1 were glucoraphanin, glucoraphenin, indole glucosinolates, and total glucosinolates. Erucic acid, isothiocyanates (sulforaphane and sulforaphene), and total fatty acids also had positive loading scores in PC1. Three-day-old (in particular) and five-day-old JTK sprouts appeared separated in plot a, indicating that they were most strongly affected by the total GLS, which was significantly higher in those sprouts than the levels found in the other sprouts.

The predominant contributor in PC 2 was total phenolics, followed closely by DPPH free radical scavenging activity. As three-day-old BSR sprouts had the highest total phenolics and among the highest AOC (both to non-significant degrees), they had the highest loading of all the three–seven-day-old sprouts tested. In fact, all three BSR sprouts loaded heavily in PC1, reflecting their high total phenolics levels and AOC. The same was true for three-day-old JSB sprouts. In fact, for all three cultivars, loading in PC1 followed the order of three > five > seven-day-old sprouts, reflecting the combined impact of total phenolics levels and AOC on the phytochemical relationship of the respective sprouts. The isothiocyanates (SFA and SFE) and erucic acid were the only analytes that had notable loading on both PC1 and PC2, indicating that they are also important determinants of the relative positioning of the cruciferous cultivars.

The strongest Pearson correlations were observed between total glucosinolates and the GSLs, glucoraphanin and glucoraphenin (0.88) and between the total phenolics and antioxidant capacity (0.86), observations that are not surprising (Table 8). Unlike what was observed for one-day-old sprouts, sulforaphane and sulforaphene correlated strongly with erucic acid (0.84), which is due to the significant reduction in the levels of both phytochemicals with sprouting duration, in general.

## 3. Materials and Methods

### 3.1. Plant Materials

Seeds of several varieties of broccoli, kale, and radishes were obtained from suppliers in Canada and the USA. Based on preliminary analysis of the seed types, three radish, one kale, and three broccoli cultivars were chosen for screening: Black Spanish Round (BSR), Miyashige (MIY), and Nero Tunda (NT) radish; Johnny’s Toscano Kale (JTK), and Johnny’s Sprouting Broccoli (JSB), Gypsy F1 (GYP), and Mumm’s sprouting broccoli (MUM). All seeds were obtained from Johnny’s Sprouting Seeds Fairfield, ME), with the exception of BSR (West Coast Seeds, Vancouver, BC, Canada), and MUM (Mumm’s Sprouting Seeds, Parkside, SK, Canada).

### 3.2. Sprouting

Approximately 1 g of fresh seeds of each cultivar was surface sterilized in 5 mL 50% bleach for 15 min, followed by rinsing thrice with copious amounts of tap water. They were then soaked in 20 mL tepid tap water (3 h for kale and broccoli seeds and 3 h and 45 min for radish seeds, which were larger), with shaking every 30 min. After soaking, the seeds were drained, patted dry with a paper towel, weighed, and sprouted in sprouting trays for 24 h in ambient fluorescent white light (16 h light/8 h dark photoperiod) and dark (covered trays) at room temperature (~22 °C), with water spraying every 4–8 h. Sprouting was replicated at different times to give three replicate samples of each sprout variety. After germination, the sprouts were weighed, then freeze-dried or oven-dried at 60 °C for 13 h prior to storage at −80 °C [9,50]. Oven-dried samples were not used for the determination of ITC contents.

### 3.3. Simultaneous Quantification of Glucosinolates and Phenolics

Dried sprouts were ground via mortar and pestle to produce a powder. Duplicate 30 mg portions were extracted with 1.0 mL of 70% methanol at 70 °C for 15 min, with vortexing for about 10 s before and after heating, followed by centrifugation at 13,000 rpm for 8 min at 4 °C. The extraction was repeated, and the combined supernatants were dried down under argon or nitrogen at 40 °C. The dried extracts were dissolved in 1 mL HPLC grade water and filtered through 0.45 µm filter, and 10 µL was analyzed on an Agilent 1100 HPLC with diode array detector and binary pump, using an XDB C18 4.6 × 150 mm 5 µm column (Agilent Technologies, Palo Alto, CA, USA). The mobile phase consisted of 0.1% trifluoroacetic acid (TFA) in water (solvent A) and 0.1% TFA in acetonitrile (solvent B), run at room temperature as reported in previous studies [22,24]. 10 µL of sample was injected, with a flow rate of 500 µL/min, and a multi-step gradient as follows: 0% B from 0–5 min; 0–17% B from 5–15 min; hold for 2 min; 17–25% B from 17–22 min; 25–35% B from 22–30 min; 35–50% B from 30–35 min; 50–99% B from 35–50 min; 99–0% B from 50–51 min; hold for 2 min; total run time, 53 min.

Glucosinolates and phenolics were respectively detected at 227 nm and 330 nm, using sinigrin monohydrate (LKT laboratories, St. Paul, MN, USA) and quercetin-3-rutinoside trihydrate (St. Louis, MO, USA) as external standards within calibration ranges of 50–2000 µM and 10–1000 µM, respectively.

### 3.4. GC-FID Analysis of Fatty Acid Contents

Gas chromatography was carried out on an Agilent 6890 instrument equipped with an autosampler and flame-ionization detector via a previously published method [51]. The column was a Varian CPSil-88 100 m × 0.25 mm with a film thickness of 0.2 µm. We extracted 30 mg sprout powder (triplicates) in the presence of C17:0 fatty acid internal standard using a modification of the method of Folch and Lees [52]. The lipid-containing phase was removed, dried under a stream of nitrogen, and methyl esters of fatty acids produced by incubation in 6% sulfuric acid in MeOH for 2 h at 80 °C. The solution was neutralized by the addition of 50% ammonium hydroxide and the fatty acid methyl esters extracted with hexane, followed by the passage of the extract through anhydrous sodium sulfate to remove traces of water. The extract was dried under a stream of nitrogen and resuspended in hexane for injection into the GC. Erucic acid (≥99%) was obtained from Sigma (St. Louis, MA, USA). Data were analyzed, and fatty acid methyl esters were quantified using commercial FAME standards (Nu-Chek Prep, Elysian, MN, USA) using Agilent Chemstation software.

### 3.5. Quantification of Isothiocyanates

The frozen, powdered sprouts (30 mg) were extracted with 700 µL distilled water by vortexing for ~10 s at room temperature (21 °C). The samples were left to autolyze over a period of 2 h, after which they were vortexed for 30 s, centrifuged at 13,000 rpm for 7 min, filtered through 0.2 µm PTFE, then stored at −80 °C prior to analysis. For quantification, 10 µL of the room temperature extracts were analysed by HPLC at 210 nm on an Agilent 1100 instrument with diode array detector and binary pump. The XDB C18 4.6 × 150 mm 5 µm column (Agilent Technologies, Palo Alto, CA, USA) was eluted at room temperature with a mobile phase consisting of 0.1% TFA in water (solvent A) and 0.1% TFA in acetonitrile (solvent B). 10 µL of sample was injected, with a flow rate of 500 µL/min, and a multi-step gradient as follows: 3–27% B from 0–20 min; 27–70% B from 20–40 min; 70–90% B from 40–41 min; hold 3 min; 90–3% B from 44–45 min; hold 5 min; total run time 50 min.

### 3.6. Determination of Total Phenolics Contents (TPC) and DPPH Free Radical Scavenging Activity

The extracts obtained for HPLC analysis of ITCs were used as well for determining the TPC and DPPH free radical scavenging activity determination. Total polyphenol content was measured using the Folin–Ciocalteu assay. Folin–Ciocalteu reagent (1:10 dilution; 75 µL) and 60 µL of 7.5% Na_2_CO_3_ were added to 15 µL of each sample solution. After 30 min of incubation in the dark, UV absorbance was determined in a microplate reader at 765 nm. The analysis was performed in triplicate, and TPC was expressed as mg gallic acid equivalents/g sample. For DPPH free radical scavenging activity, sprout extracts (10 µL) were added to 190 µL 0.4 mM DPPH solution, with subsequent incubation at room temperature (22 °C) for 30 min. The absorbance was recorded at 517 nm using a LUMIstar Omega microplate reader (BMG LABTECH, Ortenberg, Germany). Antioxidant activity was expressed as Trolox equivalence. Analyses were done in triplicates for one-day-old sprouts, and duplicates for three–seven-day-old.

### 3.7. Statistical Analysis

Values are represented as mean ± standard deviation. Data were analyzed with two-way analyses of variance (ANOVA) followed by Tukey’s multiple comparisons post-hoc tests. Statistical significance was set at *p* ≤ 0.05. Pearson’s correlation (α = 0.05) with two-tailed probability values was used to determine the correlation between the constituents of focus in the one-day-old cruciferous sprouts. Data from three–seven-day-old sprouts were analyzed by PCA in the SAS version 9.4 software package to visualize relationships among sprout cultivars and phytochemicals. Tableau version 2021.4.3 was used for creating the plots. Pearson’s correlation coefficients of the three–seven-day-old sprouts were also determined.

## 4. Conclusions

The results indicate that BSR and JTK sprouts harvested after three days are the best sources of SRE and SRA, respectively, among the cultivars analyzed. This is so, as the ITC levels were only slightly lower than those of the respective one-day-old sprouts, while they retained high levels of phenolics and AOC, and approximately 40 and 25% lower erucic acid levels, respectively, compared to one-day-old sprouts. These findings may be useful for guiding further studies on the effects of cruciferous sprouts on health, as well as product development in this area.

## Figures and Tables

**Table 1:** Glucosinolate contents in one-day-old radish, kale and broccoli sprouts grown under dark and light conditions *.

	GIB	GRA	GRE	4OHGBR	GIV	GER	GRPH	GBR	Tot. GSL	Total Indole GSL
**Retention time**	4.22	5.65	5.92	12.26	13.70	14.49	14.82	15.77		
**Dark**										
Black Spanish Round	ND	ND	76.31 ± 8.38 ^a^	5.03 ± 0.34	2.73 ± 0.49 ^ab^	ND	5.85 ± 2.21	ND	89.91 ± 6.83 ^ab^	5.03 ± 0.34
Miyashige	ND	ND	116.15 ± 8.90 ^b^	5.00 ± 0.59	2.96 ± 0.66 ^ab^	ND	12.10 ± 1.61	ND	136.21 ± 9.97 ^c^	5.00 ± 0.59
Nero Tunda	ND	ND	89.70 ± 18.16 ^a^	4.88 ± 1.21	3.37 ± 0.42 ^a^	ND	7.62 ± 4.34	ND	105.57 ± 23.46 ^ac^	4.88 ± 1.22
Toscano Kale	3.96 ± 0.62 ^a^	92.57 ± 10.61 ^a^	ND	5.14 ± 0.81	1.96 ± 0.77 ^ab^	18.53 ± 1.9 ^a^	ND	0.61 ± 0.12 ^a^	122.77 ± 13.99 ^ac^	5.75 ± 0.82
Johnny’s Sprouting Broccoli	22.22 ± 2.03 ^b^	59.08 ± 3.46 ^b^	ND	5.91 ± 0.62	1.91 ± 0.30 ^ab^	18.15 ± 2.37 ^a^	ND	0.66 ± 0.04 ^a^	107.92 ± 4.51 ^ac^	6.57 ± 0.66
Gypsy Broccoli	3.00 ± 3.72 ^a^	68.88 ± 3.72 ^c^	ND	4.78 ± 0.81	1.53 ± 0.05 ^ab^	19.73 ± 1.867 ^ac^	ND	1.89 ± 0.32 ^b^	99.8 ± 6.17 ^ac^	6.67 ± 1.12
Mumm’s Sprouting Broccoli	13.03 ± 4.95 ^c^	27.59 ± 12.86 ^d^	ND	4.42 ± 1.98	1.97 ± 0.55 ^bc^	8.45 ± 3.94 ^bc^	ND	0.64 ± 0.16 ^aA^	56.11 ± 23.35 ^b^	5.07 ± 2.13
**Light**										
Black Spanish Round	ND	ND	79.21 ± 2.17 ^a^	4.93 ± 0.39	1.9 ± 0.55	ND	3.54 ± 0.98	ND	89.81 ± 3.97 ^ac^	4.93 ± 0.39
Miyashige	ND	ND	110.34 ± 6.88 ^b^	4.74 ± 0.69	1.94 ± 046	ND	8.93 ± 2.80	ND	126.18 ± 11.08 ^bc^	4.74 ± 0.69
Nero Tunda	ND	ND	97.02 ± 2.58 ^ab^	4.57 ± 0.67	2.07 ± 1.04	ND	4.19 ± 1.48	ND	108.07 ± 4.00 ^bc^	4.57 ± 0.67
Tuscan Kale	3.69 ± 0.58 ^a^	91.18 ± 8.44 ^a^	ND	5.04 ± 0.94	1.61 ± 0.03	19.01 ± 0.78 ^a^	ND	0.65 ± 0.14 ^a^	119.87 ± 11.92 ^bc^	5.68 ± 0.95
Johnny’s Sprouting Broccoli	21.51 ± 1.28 ^b^	56.47 ± 4.36 ^b^	ND	5.82 ± 0.71	1.65 ± 0.26	16.86 ± 1.27 ^ac^	ND	0.65 ± 0.06 ^a^	102.97 ± 8.91 ^ac^	6.47 ± 0.76
Gypsy Broccoli	2.93 ± 0.26 ^c^	72.97 ± 7.46 ^c^	ND	4.98 ± 0.81	1.66 ± 0.08	20.70 ± 2.89 ^a^	ND	2.09 ± 0.18 ^b^	105.33 ± 11.29 ^ac^	7.07 ± 0.89
Mumm’s Sprouting Broccoli	16.48 ± 3.35 ^d^	35.54 ± 4.58 ^d^	ND	5.73 ± 1.04	2.18 ± 0.99	10.56 ± 1.73 ^bc^	ND	0.80 ± 0.08 ^aB^	71.29 ± 10.01 ^a^	6.53 ± 1.12

Within a column and for each lighting condition, values followed by the same lowercase letter are not significantly different (*p* ≤ 0.05). n = 3; ±SD. Within a column and for each variety, values followed by the same uppercase letter are not significantly different (*p* ≤ 0.05). n = 3; ±SD. * Progroitrin is present in trace amounts in sprouts. 4OHGBR: 4-hydroxyglucobrassicin; GBR: Glucobrassicin; GER: Glucoerucin; GIB: Glucoiberverin; GRA: Glucoraphasarin; GRE: Glucoraphenin; GIV: Glucoiverin; GSL: Glucosinolate.

**Table 2:** Pearson’s correlation coefficients among selected analyzed parameters in crucifer cultivars.

	Tot GSL	GRE/GRA	Indole GSL	SFE/SFA	Phenolics	AOC ^a^	Erucic Acid
Tot GSL	1.00						
GRE/GRA	0.94	1.00					
Indole GSL	0.09	−0.19	1.00				
SFE/SFA	0.82	0.95	−0.42	1.00			
Phenolics	0.26	0.4	−0.71	0.52	1.00		
AOC ^a^	−0.2	0.06	−0.72	0.15	0.61	1.00	
Erucic acid	−0.37	−0.56	0.51	−0.68	−0.76	−0.43	1.00

^a^ DPPH radical scavenging activity used as a measure of antioxidant activity (AOC). GRA: glucoraphanin; GRE: glucoraphanin; GSL: glucosinolate; SFA: sulforaphane; SFE: sulforaphene (percentages). In addition to numerical correlation data, correlations are classified according to a color scale, with blue indicating a positive correlation and red, a negative correlation. Color intensity indicates the strength of the correlation.

**Table 3:** Fatty acid composition of one-day-old radish, kale, and broccoli cultivars grown under dark and light conditions ^a^.

Fatty Acid	Sprout Variety						
Dark	BSR	MIY	NT	JTK	JSB	GYP	MUM
16:0	6.37 ± 0.28	6.15 ± 0.16	6.55 ± 0.20	5.63 ± 0.07	4.51 ± 0.11	4.54 ± 0.14	4.64 ± 0.41
18:0	1.41 ± 0.38	1.62 ± 0.04	1.48 ± 0.13	0.56 ± 0.04	0.28 ± 0.09	0.56 ± 0.06	0.30 ± 0.03
18:1c9	28.68 ± 0.65	28.39 ± 0.65	28.97 ± 1.11	11.00 ± 0.25	13.41 ± 0.51	18.80 ± 0.17	14.91 ± 0.06
18:1c7	1.05 ± 0.37	0.88 ± 0.09	1.38 ± 0.11	0.74 ± 0.10	0.90 ± 0.07	1.29 ± 0.13	0.78 ± 0.07
18:2	12.14 ± 0.26	10.78 ± 0.66	11.43 ± 0.29	16.84 ± 0.31	15.62 ± 0.56	15.40 ± 0.15	13.39 ± 0.55
20:0	0.63 ± 0.03	0.78 ± 0.04	0.62 ± 0.13	0.07 ± 0.03	ND	0.04 ± 0.05	0.00
20:1ω15	9.83 ± 0.31	10.15 ± 0.15	10.19 ± 0.13	3.99 ± 0.07	4.54 ± 0.30	7.96 ± 0.03	3.88 ± 0.04
18:3ω3	13.48 ± 0.51	9.18 ± 0.43	13.54 ± 0.29	12.30 ± 0.57	13.29 ± 0.68	11.87 ± 0.42	12.34 ± 0.28
20:2	0.12 ± 0.02	0.18 ± 0.04	0.00	0.32 ± 0.04	0.12 ± 0.09	0.25 ± 0.05	ND
22:0	0.26 ± 0.03	0.55 ± 0.04	0.26 ± 0.15	0.20 ± 0.05	0.19 ± 0.10	ND	ND
22:1	25.22 ± 0.42	30.80 ± 0.80	24.76 ± 1.02	47.41 ± 0.81	45.83 ± 1.01	39.16 ± 1.01	49.44 ± 1.32
22:2	ND	ND	ND	0.96 ± 0.04	0.55 ± 0.08	0.10 ± 0.07	0.31 ± 0.04
24:1	0.66 ± 0.34	0.81 ± 0.01	0.82 ± 0.11	ND	ND	ND	ND
Saturated	8.83 ± 0.33	9.10 ± 0.19	8.91 ± 0.31	6.45 ± 0.18	4.99 ± 0.06	5.16 ± 0.16	4.94 ± 0.44
MUFA	65.43 ± 0.40	70.76 ± 1.13	66.11 ± 0.45	63.13 ± 0.61	65.43 ± 1.15	67.22 ± 0.74	69.02 ± 1.23
PUFA	25.74 ± 0.71	20.14 ± 1.06	24.98 ± 0.15	30.42 ± 0.67	29.58 ± 1.13	27.62 ± 0.67	26.04 ± 0.79
**Light**							
16:00	6.35 ± 0.29	5.98 ± 0.31	6.36 ± 0.2	5.16 ± 0.21	4.55 ± 0.19	4.40 ± 0.18	4.41 ± 0.12
18:0	1.55 ± 0.13	1.52 ± 0.20	1.46 ± 0.10	0.51 ± 0.01	0.44 ± 0.11	0.50 ± 0.20	0.35 ± 0.14
18:1c9	28.15 ± 0.87	27.60 ± 0.64	28.97 ± 0.82	10.01 ± 0.69	15.28 ± 2.89	16.51 ± 3.02	14.27 ± 0.38
18:1c7	1.13 ± 0.23	0.68 ± 0.29	1.34 ± 0.20	0.59 ± 0.06	1.03 ± 0.21	1.12 ± 0.29	0.76 ± 0.04
18:2	11.76 ± 0.95	10.68 ± 0.73	11.58 ± 0.42	15.30 ± 0.96	15.10 ± 1.06	15.19 ± 0.80	13.26 ± 0.41
20:0	0.58 ± 0.14	0.67 ± 0.19	0.60 ± 0.09	0.11 ± 0.07	0.02 ± 0.01	0.13 ± 0.06	ND
20:1ω15	10.04 ± 0.54	10.21 ± 0.37	10.08 ± 0.22	3.85 ± 0.13	5.58 ± 2.00	6.71 ± 2.14	3.94 ± 0.02
18:3ω3	13.27 ± 0.84	9.35 ± 0.65	13.78 ± 0.66	11.73 ± 0.20	12.82 ± 0.73	12.68 ± 1.38	12.80 ± 0.37
20:2	0.15 ± 0.04	0.17 ± 0.04	0.12 ± 0.10	0.37 ± 0.08	0.28 ± 0.10	0.30 ± 0.19	0.09 ± 0.08
22:0	0.23 ± 0.16	0.46 ± 0.19	0.25 ± 0.09	0.28 ± 0.09	0.17 ± 0.10	0.12 ± 0.04	0.18 ± 0.10
22:1	25.96 ± 1.31	31.94 ± 2.20	24.69 ± 1.88	49.66 ± 1.91	44.22 ± 5.08	41.98 ± 4.42	49.75 ± 0.80
22:2	ND	ND	ND	1.03 ± 0.16	0.50 ± 0.36	0.37 ± 0.19	0.47 ± 0.25
24:1	0.84 ± 0.10	0.74 ± 0.13	0.79 ± 0.04	1.40 ± 0.45	ND	ND	ND
Saturated	8.70 ± 0.67	8.64 ± 0.87	8.67 ± 0.46	6.07 ± 0.05	5.18 ± 0.14	5.14 ± 0.21	4.82 ± 0.10
MUFA	66.12 ± 23.9	71.17 ± 2.21	65.86 ± 1.46	65.50 ± 0.97	66.12 ± 1.58	66.32 ± 2.05	69.25 ± 1.07
PUFA	25.17 ± 1.72	20.19 ± 1.37	25.48 ± 1.12	28.43 ± 0.92	28.69 ± 1.45	28.54 ± 2.00	25.93 ± 1.10

^a^ Fatty acid composition represents the percentage relative to total fatty acid contents. BSR: Black Spanish Round radish, MIY: Miyashige radish; NT: Nero Tunda radish; JTK: Johnny’s Toscano Kale; JSB: Johnny’s Sprouting Broccoli, GYP: Gypsy F1 radish; MUM: Mumm’s sprouting broccoli.

**Table 4:** Concentration of erucic acid and total fatty acids in one-day-old radish, kale, and broccoli sprouts.

	Concentration (mg/g DW)			
	Dark		Light	
Sprout Variety	Erucic Acid (22:1)	Total Fatty Acids	Erucic Acid (22:1)	Total Fatty Acids
BSR	16.50 ± 0.68	65.33 ± 1.95	19.28 ± 1.96	74.22 ± 5.82
MIY	18.89 ± 0.70	61.36 ± 2.29	21.59 ± 2.63	67.42 ± 3.56
NT	16.28 ± 4.70	65.75 ± 19.22	19.41 ± 3.23	78.28 ± 6.89
JTK	23.48 ± 1.05	49.55 ± 3.03	32.19 ± 7.06	63.61 ± 11.88
JSB	22.43 ± 4.05	48.47 ± 7.63	25.46 ± 4.64	57.29 ± 4.08
GYP	17.83 ± 1.51	45.59 ± 4.88	24.10 ± 1.95	58.13 ± 10.61
MUM	23.06 ± 3.68	45.76 ± 5.88	29.55 ± 8.34	57.92 ± 14.73

BSR: Black Spanish Round radish, MIY: Miyashige radish; NT: Nero Tunda radish; JTK: Johnny’s Toscano Kale; JSB: Johnny’s Sprouting Broccoli, GYP: Gypsy F1 radish; MUM: Mumm’s sprouting broccoli.

**Table 5:** Glucosinolate composition of three-, five-, and seven-day-old radish, kale, and broccoli sprouts.

		GIB	PRO	GRA	GRE	4-OHGBR	GIV	GER	GRPH	GBR		
Sprout Variety	Age of Sprouts	4.21	4.42	5.62	5.92	12.15	13.70	14.48	14.73	15.69	Tot. GSL	Total Indole GSL
BSR	3-day-old	ND	2.54 ± 1.01 ^a^	ND	32.78 ± 3.79 ^a^	3.72 ± 0.43 ^a^	3.95 ± 0.12	ND	23.35 ± 0.00 ^a^	ND	67.01 ± 5.34 ^abA^	3.72 ± 0.43 ^a^
	5-day-old	ND	6.40 ± 0.39 ^bA^	ND	22.04 ± 0.97 ^b^	2.72 ± 0.18 ^abA^	6.49 ± 0.53	ND	50.30 ± 1.06 ^b^	0.34 ± 0.00	88.96 ± 3.12 ^aA^	3.05 ± 0.19 ^ab^
	7-day-old	ND	4.31 ± 0.94 ^ab^	ND	8.34 ± 1.55 ^c^	1.23 ± 0.14 ^b^	5.24 ± 0.40	ND	13.06 ± 1.49 ^c^	0.29 ± 0.04	33.13 ± 4.59 ^bA^	1.51 ± 0.18 ^b^
JTK	3-day-old	2.87 ± 1.41 ^aA^	2.98 ± 1.18	78.83 ± 17.11 ^aA^	ND	4.11 ± 0.96 ^a^	4.04 ± 0.89 ^a^	15.83 ± 3.70 ^aA^	ND	1.20 ± 0.24 ^aA^	109.85 ± 24.47 ^aB^	5.30 ± 1.20 ^a^
	5-day-old	0.60 ± 0.85 ^bA^	3.16 ± 0.02 ^B^	18.07 ± 6.49 ^b^	ND	1.18 ± 0.39 ^bB^	5.63 ± 1.92 ^ab^	3.88 ± 0.99 ^b^	ND	0.41 ± 0.09 ^b^	32.92 ± 10.71 ^bB^	1.58 ± 0.48 ^b^
	7-day-old	2.73 ± 0.14 ^abA^	1.05 ± 0.06	77.09 ± 3.19 ^a^	ND	2.18 ± 0.02 ^bc^	8.01 ± 0.46 ^b^	13.35 ± 0.83 ^aA^	ND	1.07 ± 0.05 ^a^	105.47 ± 4.71 ^acB^	3.25 ± 0.03 ^ab^
JSB	3-day-old	9.58 ± 0.40 ^aB^	4.64 ± 0.01 ^a^	32.44 ± 1.46 ^B^	ND	3.76 ± 0.08 ^a^	3.79 ± 0.09 ^a^	9.32 ± 0.36 ^B^	ND	0.79 ± 0.05 ^B^	64.32 ± 2.29 ^AC^	4.55 ± 0.12 ^a^
	5-day-old	5.81 ± 0.38 ^bB^	9.20 ± 0.28 ^bC^	18.67 ± 2.48	ND	1.88 ± 0.04 ^b^	9.10 ± 1.34 ^a^	7.94 ± 0.14	ND	0.61 ± 0.00	53.21 ± 1.43 ^AB^	2.49 ± 0.04 ^bc^
	7-day-old	4.72 ± 0.1 ^bB^	2.51 ± 0.04 ^ac^	13.15 ± 2.38	ND	1.45 ± 0.14 ^bc^	7.33 ± 1.22 ^b^	5.67 ± 0.57 ^B^	ND	0.57 ± 0.05	35.39 ± 4.41 ^A^	2.02 ± 0.18 ^c^

Within a column and in a given cultivar, values followed by the same lowercase letter are not significantly different ((*p* ≤ 0.05). n = 2; ±SD). Within a column and on each sampling day, values followed by the same uppercase letter are not significantly different ((*p* ≤ 0.05). n = 3; ±SD). BSR: Black Spanish Round radish; JSB: Johnny’s Sprouting Broccoli; JTK: Johnny’s Toscano Kale.

**Table 6:** Fatty acid composition of Three, Five, and Seven-day radish, kale, and broccoli sprouts ^a^.

	BSR			JTK			JSB		
Fatty Acid	Day 3	Day 5	Day 7	Day 3	Day 5	Day 7	Day 3	Day 5	Day 7
16:00	7.49 ± 0.13	7.02 ± 0.30	7.40 ± 0.18	5.70 ± 0.57	5.36 ± 0.21	6.38 ± 0.36	5.09 ± 0.22	5.67 ± 0.34	5.68 ± 0.18
18:0	1.25 ± 0.05	1.27 ± 0.05	1.02 ± 0.06	0.26 ± 0.12	ND	ND	ND	ND	ND
18:1c9	23.43 ± 0.44	24.69 ± 0.68	24.55 ± 0.5	9.51 ± 0.24	9.82 ± 0.13	9.79 ± 0.35	12.64 ± 0.08	12.06 ± 0.76	11.37 ± 0.57
18:1c7	0.89 ± 0.06	0.83 ± 0.04	0.58 ± 0.05	0.47 ± 0.04	0.45 ± 0.05	0.65 ± 0.16	0.62 ± 0.18	1.06 ± 0.26	1.10 ± 0.42
18:2	12.74 ± 0.28	13.49 ± 0.36	14.02 ± 0.26	16.96 ± 0.11	17.14 ± 0.47	17.57 ± 0.54	17.41 ± 0.45	18.50 ± 0.59	17.53 ± 0.39
20:0	0.27 ± 0.04	0.34 ± 0.07	ND	ND	ND	ND	ND	ND	ND
20:1ω15	8.69 ± 0.17	8.45 ± 0.27	8.05 ± 0.23	3.28 ± 0.46	3.17 ± 0.49	2.73 ± 0.20	3.61 ± 0.12	2.76 ± 0.23	2.87 ± 0.25
18:3ω3	18.91 ± 0.48	18.62 ± 1.29	19.57 ± 0.55	15.34 ± 0.96	15.28 ± 0.91	16.42 ± 0.33	17.84 ± 0.42	21.32 ± 0.83	19.97 ± 0.93
20:2	ND	ND	ND	ND	ND	ND	ND	ND	ND
22:0	0.07 ± 0.03	0.17 ± 0.1	ND	ND	ND	ND	ND	ND	ND
22:1	26.26 ± 0.99	25.11 ± 0.79	24.67 ± 0.44	47.77 ± 1.14	48.43 ± 0.72	46.19 ± 0.26	42.79 ± 0.73	38.62 ± 1.09	41.49 ± 1.14
22:02	ND	ND	ND	0.69 ± 0.18	0.35 ± 0.45	0.27 ± 0.20	ND	ND	ND
Saturated	9.09 ± 0.11	8.80 ± 0.17	8.42 ± 0.14	5.97 ± 0.45	5.36 ± 0.21	6.38 ± 0.36	5.09 ± 0.22	5.67 ± 0.34	5.68 ± 0.18
MUFA	67.67 ± 14.89	59.09 ± 1.71	57.86 ± 1.11	61.04 ± 1.34	61.87 ± 1.14	59.36 ± 0.35	59.66 ± 0.87	54.51 ± 1.7	56.82 ± 1.49
PUFA	31.65 ± 0.74	32.11 ± 1.64	33.59 ± 0.77	32.99 ± 0.89	32.76 ± 0.94	34.26 ± 0.06	35.25 ± 0.85	39.82 ± 1.4	37.50 ± 1.32

^a^ Fatty acid composition represents the percentage relative to total fatty acid contents. BSR: Black Spanish Round radish; JSB: Johnny’s Sprouting Broccoli; JTK: Johnny’s Toscano Kale.

**Table 7:** Concentration of erucic acid and total fatty acids in three-, five-, and seven-day radish, kale, and broccoli sprouts.

		Concentration (mg/g DW)	
Sprout Variety	Age of Sprouts	Erucic Acid (22:1)	Total Fatty Acid
BSR	3-day-old	10.07 ± 0.15	38.38 ± 1.72
	5-day-old	10.44 ± 1.30	41.50 ± 3.89
	7-day-old	8.08 ± 0.41	32.75 ± 1.18
JTK	3-day-old	18.35 ± 3.98	38.36 ± 7.59
	5-day-old	16.48 ± 5.89	32.34 ± 13.33
	7-day-old	12.23 ± 1.50	25.00 ± 4.64
JSB	3-day-old	13.89 ± 1.68	32.43 ± 3.43
	5-day-old	9.03 ± 1.01	22.64 ± 3.43
	7-day-old	10.32 ± 1.32	23.32 ± 3.81

BSR: Black Spanish Round radish; JSB: Johnny’s Sprouting Broccoli; JTK: Johnny’s Toscano Kale.

**Table 8:** Pearson’s correlation coefficients among selected analyzed parameters in JSB sprouts.

	Tot GSL	GRE/GRA	Indole GSL	SFE/SFA	Phenolics	AOC ^a^	Erucic Acid	Tot FA
Tot GSL	1.00							
GRE/GRA	0.88	1.00						
Indole GSL	0.77	0.72	1.00					
SFE/SFA	0.35	0.14	0.35	1.00				
Phenolics	−0.06	−0.32	0.26	0.46	1.00			
AOC ^a^	−0.21	−0.47	0.13	0.33	0.86	1.00		
Erucic acid	0.40	0.58	0.53	0.03	−0.49	−0.30	1.00	
*Tot FA*	0.34	0.11	0.42	0.84	0.35	0.36	0.31	1.00

^a^ DPPH free radical scavenging activity used as a measure of AOC. In addition to numerical correlation data, correlations are classified according to a color scale, with blue indicating a positive correlation and red, a negative correlation. Color intensity indicates the strength of the correlation.

## Data Availability

The data presented in this study are available on request from the corresponding author.

## Abbreviations

AOC	Antioxidant capacity
BSR	Black Spanish Round (radish)
DPPH	2,2-Diphenyl-1-picrylhydrazyl
ESP	Epithiospecifier protein
FA	Fatty acid
GIB	Glucoiberverin
GIV	Glucoiverin
GRA	Glucoraphanin
GRE	Glucoraphenin
GRPH	Glucoraphasatin
GYP	Gypsy F1 (broccoli)
ITC	Isothiocyanates
JTK	Johnny’s Toscano Kale
JSB	Johnny’s Sprouting Broccoli
MIY	Miyashige (radish)
MUM	Mumm’s Sprouting Broccoli
NT	Nero Tunda (radish)
PCA	Principal component analysis
PRO	Progoitrin
SFA	Sulfuraphane
SFE	Sulfuraphene
TPC	Total phenolics contents
